# Regulatory policy and pharmaceutical innovation in the United Kingdom after Brexit: Initial insights

**DOI:** 10.3389/fmed.2022.1011082

**Published:** 2022-12-15

**Authors:** Matthias P. Hofer, Paola Criscuolo, Nilay Shah, Anne L. J. ter Wal, James Barlow

**Affiliations:** ^1^Imperial College Business School, London, United Kingdom; ^2^Department of Chemical Engineering, Imperial College London, London, United Kingdom

**Keywords:** medicines regulation, pharmaceutical innovation, United Kingdom, Brexit, health policy, Medicines and Healthcare products Regulatory Agency

## Abstract

Brexit was presented as an opportunity to promote innovation by breaking free from the European Union regulatory framework. Since the beginning of 2021 the Medicines and Healthcare products Regulatory Agency (MHRA) has operated as the independent regulatory agency for the United Kingdom. The MHRA's regulatory activity in 2021 was analyzed and compared to that of other international regulatory bodies. The MHRA remained reliant on EU regulatory decision-making for novel medicines and there were significant regulatory delays for a small number of novel medicines in the UK, the reasons being so far unclear. In addition, the MHRA introduced innovation initiatives, which show early promise for quicker authorization of innovative medicines for cancer and other areas of unmet need. Longer-term observation and analysis is needed to show the full impact of post-Brexit pharmaceutical regulatory policy.

## Introduction

Pharmaceutical innovation helps to improve the efficacy of healthcare systems and population health outcomes (1–3). Global expenditure on pharmaceuticals reached approximately 1.27 trillion U.S. dollars in 2020 and has been forecasted to further increase to 1.6 trillion by the year 2025 (4). On average this accounts for approximately 15% of all healthcare spending in 2020 in OECD countries (5). The development of pharmaceutical innovation is a global industry, but the location of research and development (6), economic benefits (7), and access to pharmaceutical innovation (8, 9), are not equally spread across the world. Regional and national policy interventions can be important tools to stimulate and foster R&D investment in the pharmaceutical industry and improve access to new medicines. Pharmaceutical regulatory policy can impact development and access to pharmaceutical innovation by providing incentives and offering regulatory flexibility (10).

In the United Kingdom (UK), the pharmaceutical industry (including biopharma) employed 66,000 people (11), and invested approximately £ 5 billion in R&D in 2020 (12). Prior to the exit from the European Union (EU), the national pharmaceutical regulatory policy of the UK was shaped by the European Union (EU) and the UK medicines regulator Medicines and Healthcare products Regulatory Agency (MHRA) was an active member of the European Medicines Agency (EMA) (13). Brexit was presented as an opportunity to promote innovation by breaking free from the EU regulatory framework for medical technologies. After the EU-exit vote and during the time of EU-UK negotiations, there were numerous reflections by experts and the pharmaceutical industry highlighting a potential risk of delay or lack of access to pharmaceutical innovation, amid an increased regulatory burden due to the need to file separate drug authorizations for the UK (13–15). Furthermore, it was feared that the willingness of pharmaceutical industry to launch new drugs in the UK could be hampered by the UK's relatively small share of the global pharmaceutical market (2.4%) (16, 17). Since the beginning of 2021 the MHRA has operated as the stand-alone and independent regulatory agency for medicines and medical devices for the UK. The UK government and the MHRA published policy documents which set out a vision for a sovereign and independent regulatory system for medicines that is innovative in its regulatory processes, delivers rapid regulatory assessments, capitalizes on national and international cooperation, and promotes access to pharmaceutical products as early as possible (18, 19). In its response, the UK pharmaceutical industry, represented by the Association of the British Pharmaceutical Industry (ABPI), also advocated for an internationally competitive regulatory framework with a focus on innovation (16).

The objective of this study was to analyze the first year of independent regulatory activity by the MHRA in the context of the new vision for pharmaceutical innovation as set out by the UK government and compare it to that of other international regulatory agencies in USA (FDA), EU (EMA) and Switzerland (Swissmedic).

## Methodology

Pharmaceutical innovation was defined as medicinal products containing new active substances. This is a commonly used measure for pharmaceutical innovation (20–23), but it may not fully capture the level of innovation in terms of intrinsic pharmacological properties (24) or therapeutic value for patients (25, 26). Publicly available assessment reports were used to establish the level of product innovation by using the framework developed by Ferner and Aronson, which takes into account the medicine's structure/composition, target, clinical use, safety, delivery, and quality (24, 27).

Data on medicines approvals in 2021 were collected from public repositories provided by regulatory agencies (28–33). The analyses presented here only concern medicinal products that were approved in 2021 by any of the agencies. This defined set of medicines has been tracked across all jurisdictions until 1. August 2022, which was the end of the follow-up period for this analysis.

For international comparisons, the number of all novel medicines was adjusted to exclude diagnostics, vaccines including COVID-19 vaccines, COVID-19 therapeutics, source plasma products, and medicinal products that were associated with negative appraisals/withdrawals, generics, or reference approvals for other countries/regions.

For drug approval lag analysis, all novel medicines by each respective agency were pooled and drug authorization lag was determined between the day of first authorization by any of the four agencies and the date of subsequent authorization by the other agencies. From this pool of novel medicines, approximately half were found to be approved by all four agencies (45/97, 46%) at the end of the follow-up period, hence the analysis cut-off date of 1 August 2022 was imputed when an authorization date was missing. Analysis without imputation has been conducted and provides the same pattern of results (data not shown).

## Pharmaceutical innovation in the UK

The analysis of the first year of regulatory independence found that MHRA granted a total of 1,374 licenses for 561 medicinal products. The majority of licensing activity concerned medicines with known active substances, but 44 medicinal products (8%) were novel and contained new active substances.

**Figure 1:**
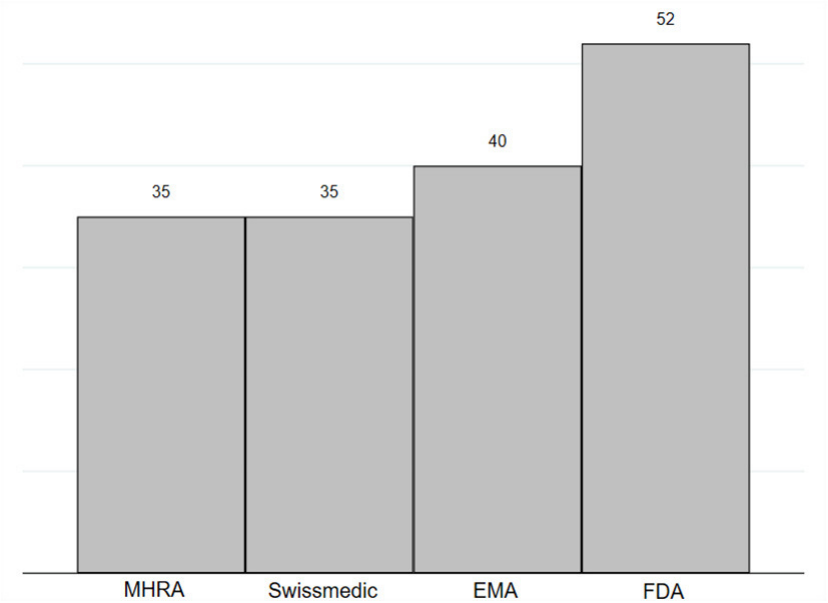
Number of novel medicines authorized in 2021 by MHRA, EMA, FDA and Swissmedic. Novel medicines were defined as medicines with new active substance (*n* = 97). In this figure displays data published by FDA on New chemical entity (NCE) and Biologics License Applications (BLA), by EMA on New Active Substance (NAS), and by Swissmedic on authorized human medicines with new active substances (29–32). MHRA does not publish similar data and new active substance status was assessed on the basis of the assessment report by study authors (28, 33). Figures were adjusted to exclude diagnostics, vaccines including COVID-19 vaccines, COVID-19 therapeutics, diagnostics, source plasma products, and medicinal products that were associated with negative appraisals/withdrawals, generic approvals, or reference approvals for other countries/regions.

International comparison with the regulatory activity of FDA, EMA, and Swissmedic was conducted on an adjusted number of all novel medicinal products of 2021. FDA authorized 52 (65 unadjusted) novel medicines in 2021, which was found to be the highest figure amongst the analyzed regulators. EMA authorized 40 (50 unadjusted) and MHRA and Swissmedic lagged behind with the approval of 35 novel medicines in 2021 (44 unadjusted for MHRA, 43 unadjusted for Swissmedic, see Figure 1).

Five medicines had EU authorization in 2021 without equivalent MHRA approval (see Table 1). When analyzing the type and level of product innovation as previously established by Ferner and Aronson (24, 27), four medicines can be categorized as highly innovative with novel mechanisms of action, targeting rare diseases according to regulatory definitions of EU and UK, and/or fulfilling an unmet clinical need. During the analysis follow-up period, three of the medicines remained unapproved in the UK and two of the medicines were authorized with significant delay (see Table 1).

**Table 1:** Novel products authorized by EMA in 2021 with lack or delay in authorization by MHRA.

**Product** **(Active substance)**	**Disease**	**MHRA** **date**	**EMA date**	**Product innovation characteristics**	**Company (Headquarter)**
Evkeeza (evinacumab)	Homozygous familial hypercholesterolaemia (Rare disease)	-	17/06/2021	Highly innovative • Novel mechanism of action in the disease • First-in-class medicine	Regeneron (Ireland)
Enspryng (satralizumab)	Neuromyelitis optica spectrum disorders (Rare disease)	-	24/06/2021	Highly innovative • Novel mechanism of action in the disease	Roche (Switzerland)
Abecma (idecabtagene vicleucel)	Multiple myeloma (Rare disease)	24/06/2022	18/08/2021	Highly innovative • Novel mechanism of action in the disease • Gene therapy	Bristol-Myers Squibb (USA)
Voxzogo (vosoritide)	Achondroplasia (Rare disease)	-	26/08/2021	Highly innovative • Novel mechanism of action in the disease • First medical treatment for the disease	BioMarin (USA)
Artesunate Amivas (Artesunate)	Malaria	29/04/2022	22/11/2021	Slightly innovative • Already recommended by WHO • No change in health or non-health related properties	Amivas (USA)

Table includes medicines containing a new active substance authorized by EMA in 2021 that had a delay or lack of marketing authorization by the MHRA at the end of the analysis follow-up (1 August 2022).

The underlying reasons for the observed regulatory delays are currently unclear and cannot yet be conclusively elucidated from the gray literature and publicly available data by MHRA. From the perspective of the MHRA, differences in authorization dates could be caused by regulatory differences in submission and scientific assessment, but MHRA currently does not provide procedural information on their ongoing and finalized assessments. From the perspective of the applicant, diverse downstream factors might play a role, including manufacturing considerations, marketing strategies, and/or policies on patient access and healthcare spending. For example, the developer of idecabtagene vicleucel for Multiple Myeloma faced manufacturing issues after initial approval in the USA (34). Another example is vosiritide, which is the very first pharmaceutical intervention in achondroplasia that has been launched in US, France and Germany (35). However, doubts have been expressed by experts and patients about vosoritide's long-term clinical benefit and effect on quality of life (36, 37) and England's health technology assessment agency NICE's pre-authorization scoping technology appraisal for vosoritide is currently on hold until 2023 (38). Approaches by different health technology appraisal authorities in different jurisdictions might therefore influence the willingness to seek regulatory approval and uptake. Indeed, this was confirmed by an article in the Financial Times in which a pharmaceutical company outlined that they were not seeking UK approval in the belief that the NHS would not reimburse the product for NHS patients (39).

## Drug approval timing and EU reliance

**Figure 2:**
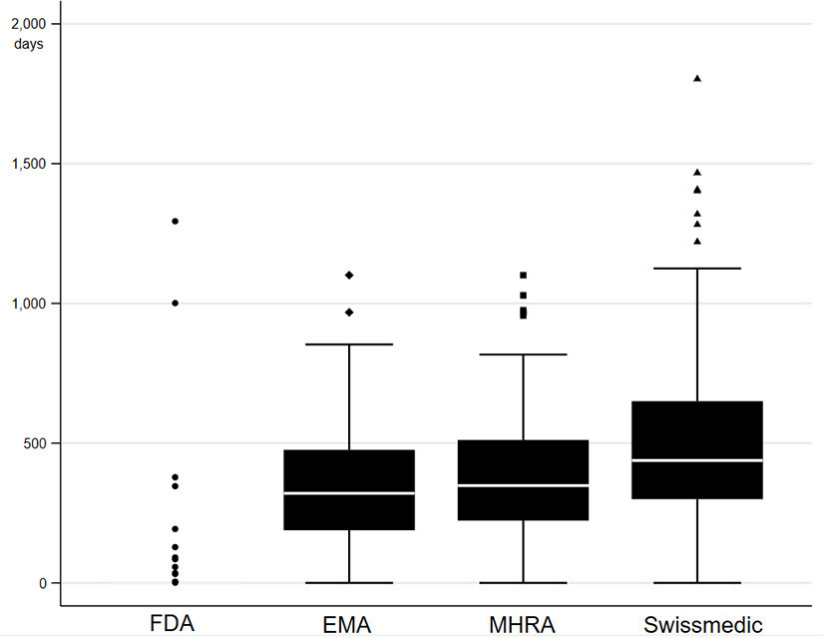
Drug approval lag in days for novel medicines authorized in 2021. A pool of medicines was created by adding all novel medicines in 2021 as declared by each respective agency considering the same adjustments discussed above (29–31, 33). The pool contained in total 97 medicines, of which 96 were authorized by FDA, 70 were authorized by EMA, 67 were authorized by MHRA, 52 were authorized by Swissmedic, and 45 were authorized by all four agencies. Drug approval lag was defined as the number of days between the first date of authorization by any of the four agencies to date of authorization by each agency. Authorization date was imputed with final day of analysis follow-up (1 August 2022) in case authorization date was not yet available. One outlier was removed, which was characterized by an approval lag above 3,000 days for EMA, MHRA and Swissmedic compared to FDA.

Drug approval lag analysis was conducted to benchmark regulatory performance and identify potential delays (see Figure 2). FDA was found to be the fastest, having approved 86% (n = 84) of novel medicines prior to all other agencies between 2012 and 2021. EMA and MHRA had relatively similar median approval lags in authorization, with 320.5 days and 348 days respectively. Swissmedic was the slowest, with a median authorization lag of 438 days. This analysis could not elucidate if the drug approval lag was caused by delays associated with MHRA submission and appraisal due to the lack of publicly available data by the MHRA, so it remains unclear if the approval lag is attributable to agency performance or alternatively to commercial barriers for industry. While these figures only represent a short-term view for the UK after Brexit, the results for USA, EU and Switzerland are similar to previous long-term studies looking at international approval gaps (40–42).

EU reliance procedures might explain why the MHRA drug approval lag remained largely in line with that of the EMA in 2021. With the beginning of independence in 2021, MHRA introduced a new EU reliance procedure for a limited time of 2 years to allow for less burdensome and more streamlined authorizations that take into consideration EU decision making. An analysis was conducted into the extent of EU reliance by screening publicly available MHRA public assessment reports for the type of authorization (33). At the time of the analysis at the end of 2021, 358 public assessment reports had been published for the assessment of 561 medicinal products. Assessment of these available reports suggests that MHRA made use of the EU reliance mechanism for 75 (21%) products. MHRA was more mostly reliant on EU regulatory decision making in the case of novel medicinal products, where 24 of 31 products (68%) were authorized *via* EU reliance procedures. These figures might not satisfy the ambitious Brexit vision for independent regulatory decision making (19), but EU reliance at least temporarily ensures regulatory stability at a time of political uncertainty and organizational changes partly resulting from MHRA re-organization and staff reductions (18, 43). Furthermore, regulatory stability can be seen to be in the interest of UK's biopharma industry because it offers greater regulatory certainty across large areas of the world. The ABPI has also called for alignment with other international regulators on wider international standards of basic life science regulation where possible (16).

The currently available data indicate that UK approval timing will largely stay in line with the time of EU approval as long as the reliance procedure remains in place. The EU reliance procedures are supposed to end in 2023, when the monitoring of the drug approval lag compared to EU and other international regulators will be of higher interest. The comparison between MHRA and Swissmedic might be of particular interest, given that Swissmedic is in a similar strategic position as an independent European regulatory agency without formal regulatory alignment to the EU but participating in the same international collaborations.

## MHRA's initiatives for pharmaceutical innovation

In light of concerns that the approval of pharmaceutical innovation might slow-down in a stand-alone regulatory system that is no longer collaborating with the EU, the UK Life Sciences vision aims to compensate by setting out a progressive UK regulatory offer with innovative regulatory processes and the opportunity to cooperate with other “likeminded” regulators (19). Since Brexit, MHRA has enhanced its pre-authorization incentives and early approval initiatives and has initiated new international collaborations beyond the EU to foster pharmaceutical innovation. These initiatives include the “Innovative Licensing and Access Pathway” (ILAP) the “Promising innovative medicine/Early Access to Medicines Scheme” (EAMS), and membership of the “Access Consortium”, and “Project Orbis”.

ILAP was created to support and accelerate the development of novel medicines and repurposed medicines in life-threatening or seriously debilitating conditions with significant unmet need. It allows flexible support tools through the life cycle of medicines development using a multi-agency approach that include MHRA, HTA bodies, the National Health Service, and other agencies that support clinical research (44).

The Access consortium is a network of regulatory authorities which aims to foster collaboration, regulatory alignment, and capacity building between the partner agencies that include Therapeutic Goods Administration (Australia), Health Canada (Canada), Health Sciences Authority of Singapore and Swissmedic. This is mainly facilitated *via* regular meetings and working groups (45).

EAMS is a national initiative for medicines for life threatening or seriously debilitating conditions when there is a clear unmet medical need. It provides an early access authorization, which can be used by physicians for off-label prescribing before formal MHRA approval. Four medicines have been granted EAMS authorization in 2021 (46) (see Table 2), but despite the scope of the scheme only two of these (in oncology) were classified as highly innovative according to Ferner and Aronson's framework (24, 27). The other two medicines were determined to be slightly innovative, given the existence of alternative interventions (in atopic dermatitis and Pompe disease). Nevertheless, the system was effective in providing interim early access when comparing the EAMS early access date to formal authorization dates of FDA, EMA and Swissmedic for these medicines. EAMS authorization was consistently quicker compared to EMA approval and in two cases also led to faster approval compared to FDA. The benefit of early access was less substantiated when considering the date of full MHRA approval of the four medicines.

**Table 2:** MHRA products authorized *via* innovation initiatives in 2021.

**Product** **(Active substance)**	**Disease**	**Product innovation characteristics**	**Company (Headquarter)**	**Initiative**	**UK regulatory approval by type** **Date** **(timing relative to initial EAMS approval in days)**		**International regulatory approval** **Date** **(timing relative to full MHRA approval in days)**		
					**Initial EAMS**	**Full MHRA**	**FDA**	**EMA**	**Swissmedic**
Pemazyre (pemigatinib)	Cholangiocarcinoma (Rare disease)	Highly innovative • Novel mechanism of action in the disease • Targeted therapy for cancer with specific genomic alterations	Incyte Biosciences (USA)	EAMS	15/01/2021	07/04/2021 (+ 82)	17/04/2020 (−355)	26/03/2021 (−12)	13/07/2021 (+97)
Cibinqo (abrocitinib)	Atopic dermatitis	Slightly innovative • Alternative Jak inhibitor • No new class of medicine or novel mechanism • No fewer adverse reactions or other therapeutic advantages	Pfizer (USA)	EAMS	28/01/2021	08/09/2021 (+223)	14/01/2022 (+128)	09/12/2021 (+102)	05/04/2022 (+209)
Nexviadyme (avalglucosidase alfa)	Pompe disease (Rare disease)	Slightly innovative • Alternative enzyme replacement therapy • No new class of medicine or novel mechanism • No fewer adverse reactions or other therapeutic advantages	Sanofi (France)	EAMS	05/03/2021	06/07/2022 (+488)	06/08/2021 (−334)	14/06/2022 (−22)	17/11/2021 (−231)
Tepmetko (tepotinib)	Non-small cell lung cancer	Highly innovative • Novel mechanism of action in the disease • Targeted therapy for cancer with specific genomic alterations	Merck KGaA (Germany)	EAMS Project Orbis	12/07/2021	24/09/2021 (+74)	03/02/2021 (−233)	16/02/2022 (+145)	22/06/2021 (−94)
Trodelvy (sacituzumab govitecan)	Breast cancer	Highly innovative • Novel mechanism of action in the disease • First-in-class medicine • Unmet clinical need in target patient population	Gilead (USA)	Project Orbis	-	08/09/2021	22/04/2020 (−504)	22/11/2021 (+75)	09/09/2021 (+1)
Lumykras (sotorasib)	Non-small cell lung cancer	Highly innovative • Novel mechanism of action in the disease • First-in-class medicine • Unmet clinical need in target patient population First-in-class medicine	Amgen (USA)	Project Orbis	-	08/09/2021	28/05/2021 (−103)	06/01/2022 (+120)	16/12/2021 (+99)
Rybrevant (amivantamab)	Non-small cell lung cancer	Highly innovative • Novel mechanism of action in the disease • Unmet clinical need in target patient population	Janssen/ Johnson & Johnson (USA)	Project Orbis	-	15/11/2021	21/05/2021 (−178)	09/12/2021 (+24)	20/01/2022 (+66)

Table includes medicines containing a new active substance authorized by MHRA *via* the Early access to medicines scheme (EAMS) scheme and/or *via* Project Orbis. Displayed are the product characteristics and approval dates by MHRA (EAMS date and full MHRA approval date), EMA, FDA and Swissmedic. Furthermore, the timing of approval is displayed relative to the full MHRA approval date. The analysis cut-off date was 1 August 2022.

Project Orbis is an international initiative with the objective of faster approval for promising cancer treatments. It is coordinated by the FDA, and as well as the USA it also involves the UK, Australia, Canada, Singapore, Switzerland, and Brazil. Four novel highly innovative oncology medicines were authorized by MHRA *via* this scheme in 2021 (47), providing consistently faster approval times than the EMA (see Table 2). The other comparator agencies are also members of Project Orbis and FDA was the fastest agency overall.

## MHRA's initiatives during COVID-19

The UK Life Sciences Vision presented the response to COVID-19 as another prominent example for regulatory independence and agility (19). Indeed, MHRA has shown willingness to enable quick access to COVID-19 vaccines without formal regulatory approval. It triggered exceptional temporary use authorizations in line with article 174 of The Human Medicines Regulations 2012, which allows for temporary distribution in response to the suspected or confirmed spread of pathogenic agents (see Table 3). This same preparedness was not shown by EU member states, which were in theory also allowed to trigger this legal exception under EU law (DIRECTIVE 2001/83/EC) but preferred to wait for centralized decision making by EMA. This led to delays in approval of the first two main COVID-19 vaccines by Pfizer/Biontech and AstraZeneca in Europe of 19 days and 30 days respectively (29, 33). Regarding COVID-19 therapies, EMA has authorized three medicines that have not been authorized by the MHRA including the two repurposed active substances anakinra and tocilizumab and the neutralizing antibody therapy regdanvimab. Nevertheless, MHRA was faster in authorizing the first newly developed antiviral medicines molnupiravir and PF-07321332 (see Table 3) (29, 33). It should be noted that there remains controversy amongst worldwide regulators regarding the clinical evidence base of molnupiravir, casting doubts about a future EU authorization (48). This highlights the importance of a balanced approach to regulatory assessment during a global pandemic by taking into consideration clinical evidence and earliest feasible patient access to medical innovation.

**Table 3:** COVID-19 interventions authorized by MHRA and EMA in 2020/21.

**Product type**	**Product name**	**Active substance**	**MHRA authorization date**	**EMA authorization date**
Vaccine	Comirnaty	Vaccine Biontech/Pfizer	02/12/2020 *	21/12/2020
	JCOVDEN	Vaccine Janssen	28/05/2021	11/03/2021
	Nuvaxovid	Vaccine Novavax	03/02/2022	20/12/2021
	Spikevax	Vaccine Moderna	08/01/2021**	06/01/2021
	Vaxzevria	Vaccine Astrazeneca	30/12/2020 ***	29/01/2021
Antiviral medicines & neutralizing antibody therapies	Regkirona	Regdanvimab	-	12/11/2021
	Ronapreve	Casirivimab / imdevimab	19/08/2021	12/11/2021
	Xevudy	Sotrovimab	01/12/2021	17/12/2021
	Veklury	Remdesivir	03/07/2020	03/07/2020
	Lagevrio	Molnupiravir	04/11/2021	-
	Paxlovid	PF-07321332 and ritonavir	31/12/2021	28/01/2022
Anti-inflammatory medicines	Kineret	Anakinra	-	17/12/2021
	RoActemra	Tocilizumab	-	06/12/2021

Table includes vaccines, antiviral therapies and anti-inflammatory therapies that have been authorized by either MHRA, EMA or both. Displayed are the dates of formal approval by MHRA and EMA, the analysis cut-off date was 1 August 2022. ^*^Temporary use (Article 174), formal authorization on 09/07/2021, ^**^Temporary use (Article 174), formal authorization on 31/03/2021, ^***^Temporary use (Article 174), formal authorization on 24/06/2021.

## Conclusions

This initial analysis on the first year of independent regulatory activity by the MHRA is naturally limited by a short observation time, a short follow-up time, and by a relatively small dataset. Furthermore, the publicly available evidence is currently insufficient to fully elucidate if any observed delays are due to differences in regulatory submission and decision making or other up- or down-stream factors in drug development, approval or access. MHRA and UK policy makers should consider providing a higher level of transparency and more timely publication of regulatory activity on the authorization of novel medicines.

As a result of these limitations, it is impossible to draw conclusions yet about the relative importance of any observed regulatory delays in the introduction of pharmaceutical innovation or on patient access and population health. The latter is further influenced by the adoption of novel medicines by health technology agencies, payers, and/or buyers from the healthcare service. However, this analysis provides the first evidence on the regulatory activity of MHRA in light of the post-Brexit regulatory vision. The UK government and the MHRA published policy documents which set out a vision for a sovereign and independent regulatory system for medicines that is innovative, rapid, and capitalizes on national and international cooperation (18, 19). MHRA has indeed been successful in providing innovative regulatory mechanisms that are partially built on international collaborations; these have shown early promise for quicker approval of more innovative medicines for cancer and other areas of unmet clinical need. Furthermore, MHRA has shown agility in the rapid approval of medicines in the interest of public health, i.e., COVID vaccines and therapeutics. With the introduction of the EU reliance procedures, MHRA lost some of its sovereignty and independence but on the other hand provided regulatory stability that ensured the authorization of most novel medicines without significant delays during a challenging time characterized by a global pandemic, political uncertainty and organizational restructuring.

Nevertheless, this analysis also presents preliminary findings that there could be an emerging risk of delays in authorization of some novel medicines compared to the EU, despite the EU reliance procedure. Only longer -term observation and analysis will be able to show the full impact of post-Brexit pharmaceutical regulatory policy and the regulatory activity of an independent MHRA on medicines availability, population health, and the UK biopharmaceutical industry.

## Author contributions

MH developed the initial idea for this article, and was responsible for data collection, data analysis, and drafting the manuscript. All authors contributed to the final manuscript and conclusions.
